# Progesterone Treatment Attenuates Glycolytic Metabolism and Induces Senescence in Glioblastoma

**DOI:** 10.1038/s41598-018-37399-5

**Published:** 2019-01-30

**Authors:** Fahim Atif, Seema Yousuf, Claudia Espinosa-Garcia, Elena Sergeeva, Donald G. Stein

**Affiliations:** Brain Research Laboratory, Department of Emergency Medicine, School of Medicine, Emory University, Atlanta, GA 30322 USA

## Abstract

We examined the effect of progesterone treatments on glycolytic metabolism and senescence as possible mechanisms in controlling the growth of glioblastoma multiforme (GBM). In an orthotopic mouse model, after tumor establishment, athymic nude mice received treatment with progesterone or vehicle for 40 days. Compared to controls, high-dose progesterone administration produced a significant reduction in tumor size (~47%) and an increased survival rate (~43%) without any demonstrable toxicity to peripheral organs (liver, kidney). This was accompanied by a significant improvement in spontaneous locomotor activity and reduced anxiety-like behavior. In a follow-up *in vitro* study of U87MG-luc, U87dEGFR and U118MG tumor cells, we observed that high-dose progesterone inhibited expression of Glut1, which facilitated glucose transport into the cytoplasm; glyceraldehyde 3-phosphate dehydrogenase (GAPDH; a glycolysis enzyme); ATP levels; and cytoplasmic FoxO1 and Phospho-FoxO1, both of which control glycolytic metabolism through upstream PI3K/Akt/mTOR signaling in GBM. In addition, progesterone administration attenuated EGFR/PI3K/Akt/mTOR signaling, which is highly activated in grade IV GBM. High-dose progesterone also induced senescence in GBM as evidenced by changes in cell morphology and β-galactocidase accumulation. In conclusion, progesterone inhibits the modulators of glycolytic metabolism and induces premature senescence in GBM cells and this can help to reduce/slow tumor progression.

## Introduction

With a dismal prognosis, glioblastoma multiforme (GBM; WHO grade IV) has the highest incidence of all malignant brain tumors. An estimated 12,390 new cases were predicted in 2017 in the US alone^1^. The aggressive nature of GBM is due to its widespread invasiveness, the difficulty of achieving complete resection, and its resistance to chemo and radiation therapy. Despite currently “optimal” treatment regimens, which include neurosurgery, radiation and temozolomide chemotherapy, the median survival of patients diagnosed with GBM is only 12–15 months^2^. GBMs recur in virtually 100% of cases, and treatments for recurrence are largely ineffective^2^.

Interestingly, GBMs appear to be more common in males, who also tend to have worse clinical outcomes than females^3,4^. Data from the Cancer Genome Atlas data set suggests that specific GBM subtypes (mesenchymal and neural) are more common in men^5^. The role of gender-specific hormones in GBM development and progression is controversial and poorly understood, so further study is warranted, especially since therapeutic strategies may emerge that are related to hormonal factors which can influence tumor growth and persistence in the face of standard treatments.

Progesterone (Pregn-4-ene-3, 20-dione) is a pleiotropic neurosteroid hormone reported to exert anti-tumor effects in some forms of cancer. High natural progesterone levels during pregnancy are essential for well-controlled fetal growth for normal development and are associated with a lower incidence of maternal breast and ovarian cancer and a long-term protective effect against some cancers^6,7^. We previously reported that treatment with high-dose progesterone reduces GBM growth and prolongs survival in mice with U87MG malignant glioma subcutaneous xenografts^8^, and it outperforms temolozolomide treatment in limiting the growth of human GBM cells *in vitro*^9^. However, other reports have not found positive effects. Some of the negative findings may be attributable to low dosing and to the use of synthetic progestins, which do not have the same receptor binding and cellular metabolic consequences as natural progesterone^10^.

Metabolic reprogramming and resistance to cell death are hallmarks of cancer. Although they can support mitochondrial glucose oxidation, cancer cells prefer to metabolize glucose to lactate. This process generates less ATP per molecule of glucose, but cancer cells derive significant gain in overall ATP production from this metabolic shortcut^11^. Metabolic reprogramming is highly dynamic and not only supports cancer cells’ high anabolic activity for uncontrolled proliferation, but also exacerbates their migrative, invasive and metastatic properties^12,13^. In general, due to the increasing energy demand of rapidly growing tumors, tumor cells undergo temporary or sustained hypoxic conditions, leading to the cancer cell’s preference for glycolysis^14^. The principal cellular mechanism for such biological adaptation in response to hypoxia is the stabilization of Hypoxia-Inducible Factor (HIF). Stabilized HIF-1α inhibits mitochondrial biogenesis and functionally cooperates with the c-Myc oncogene to promote glycolytic metabolism^15^. This includes the up-regulation of glucose transporter isoform 1 (Glut1), which allows an increase in glucose uptake. GBM is highly metabolically active and there are reports suggesting that Glut1 metabolism is upregulated in GBM cells^16^.

It is known that PI3K/Akt/mTOR signaling is highly active in GBM cells, where it supports proliferation, invasiveness and resistance to cell death mechanisms. The mammalian target of rapamycin (mTOR) is also reported to control glycolytic metabolism in GBM through the forkhead box class O1 (FoxO1) transcription factor and c-Myc upregulation in an Akt-independent manner^17^. HIF-1a, c-Myc, mTOR and FoxO1 are among the critical regulators of metabolic reprogramming in rapidly growing cancer cells^14,17^. A growing literature suggests targeting metabolic regulators in tumor cells as a potential treatment strategy^18,19^.

In this study, we tested the hypothesis that high-dose progesterone treatment will inhibit GBM growth and prolong survival by blocking glycolytic metabolism and by inducing senescence in the tumor without causing significant side effects. We examined the anti-tumor efficacy of high-dose progesterone in an orthotopic GBM mouse model and evaluated the effect of progesterone on the metabolic regulators mTOR, FoxO1, Glut1, GAPDH (a glycolytic enzyme), ATP levels as an outcome of metabolic changes, and PI3K/Akt/mTOR signaling as possible mechanisms/pathways of progesterone action in GBM cells. Because some reports suggest that low-dose progesterone may exert a proliferative effect in different tumor cell lines^8,9^, we also tested the effect of a low dose of progesterone on GBM growth *in vivo*.

## Results

### Luciferase assay and effect of progesterone on U87MG-luc viability

**Figure 1:**
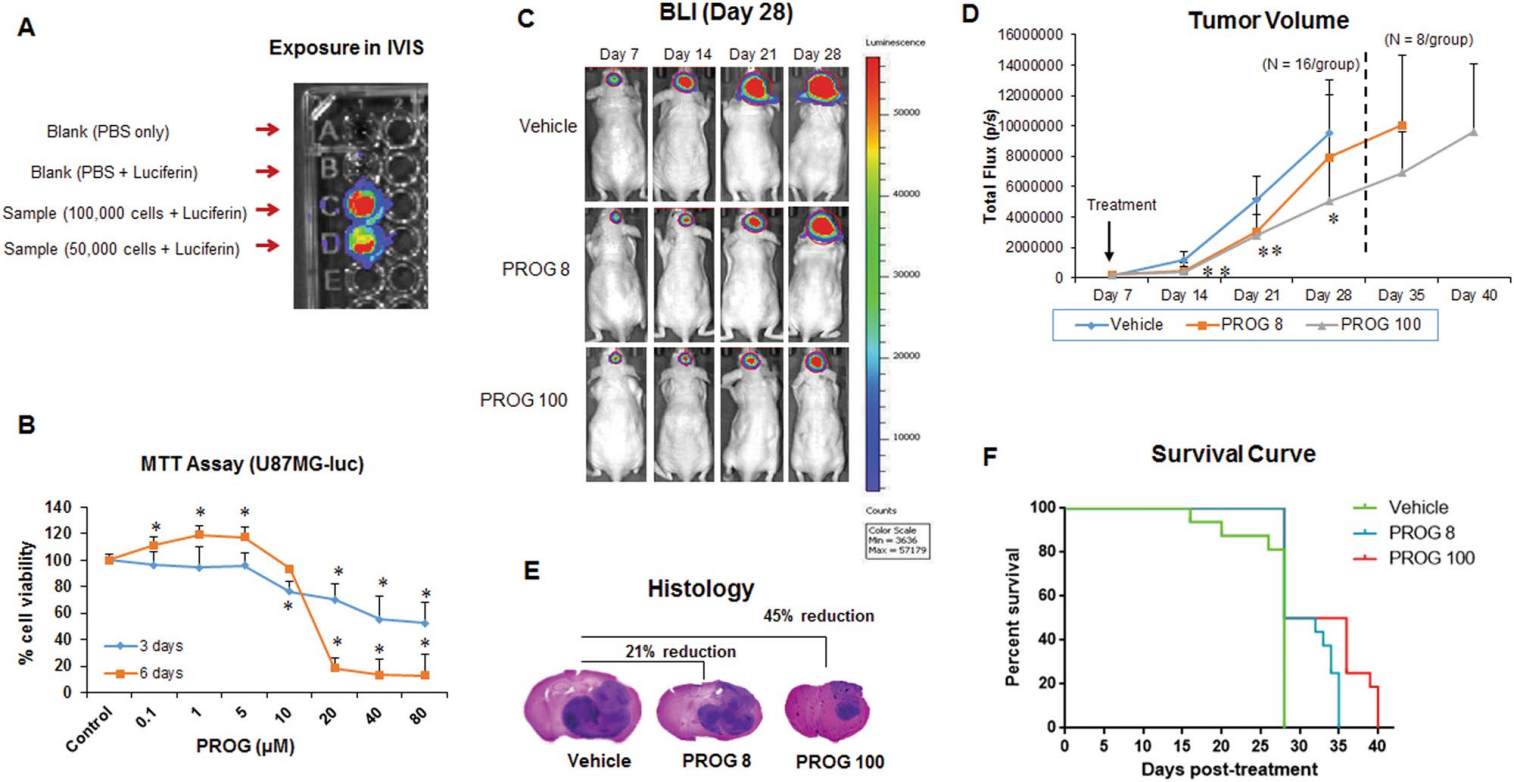
Effect of progesterone on tumor growth and survival rate. (**A**) Luciferase assay; (**B**) MTT reduction assay in U87MG-luc cells (n = 6 each); (**C**) representative images of BLI; (**D**) tumor volume (**E**) brain-tumor histology (H&E); and (**F**) survival rate of tumor-bearing mice in different groups (n = 16 each). Values are expressed as mean ± SD in different groups. *P < 0.01: Significant difference compared to control/vehicle group.

U87MG-luc cells were cultured and tested for stable luciferase activity by luciferase assay before being injected into the mice (Fig. 1A). For the *in vitro* study, we tested the effect of progesterone on the viability of U87MG-luc cells. MTT assay showed significant (*p* < 0.05) cell death in U87MG-luc cells after just 3 days of progesterone exposure at high concentrations (10, 20, 40, and 80 µM), whereas lower concentrations of progesterone (0.1, 1, 5 µM) did not induce any cell death (Fig. 1B). This cell-death-inducing effect was more pronounced after 6 days of exposure than after 3 days. Also as expected, we observed a significant (*p* < 0.05) proliferative effect of progesterone during the 6 days of exposure at the lower concentrations (0.1, 1, 5 µM).

### Effect of low- and high-dose progesterone on GBM growth and survival rate

We tested the hypothesis that high-dose progesterone would inhibit the growth of intracranial GBM. After confirming tumor establishment at day 7 post-inoculation, mice were injected with progesterone at low (PROG8) and high (PROG100) doses or vehicle daily for 3 weeks. The tumor growth was monitored and quantified by BLI every week. Repeated measures ANOVA on tumor volume revealed a significant group effect following progesterone treatments (F_(2,45)_ = 9.82; *p* < 0.001). On day 28 post-inoculation, BLI revealed a significant (*p* < 0.05) decrease in tumor growth in the PROG100 group (~47%) compared to the vehicle group (Fig. 1C,D). Interestingly, a relatively low dose of progesterone (PROG8) also showed some inhibition of tumor growth (~17%) compared to the vehicle group at day 28, but it was not statistically significant. Brain-tumor histology also showed a significant reduction in the tumor size in PROG100 (~45%) and PROG8 (~21%) groups compared to the vehicle group (Fig. 1E). For the *in vivo* survival study, half of the animals from both progesterone-treated groups continued to receive progesterone injections until they reached the maximum tumor burden or died. PROG100 treatment significantly (*p* < 0.05) enhanced the survival time of tumor-bearing mice by ~43% compared to vehicle-treated mice (Fig. 1F).

### Effect of progesterone on tumor proliferation, angiogenesis, apoptosis and PI3K/Akt/mTOR signaling *in vivo*

**Figure 2:**
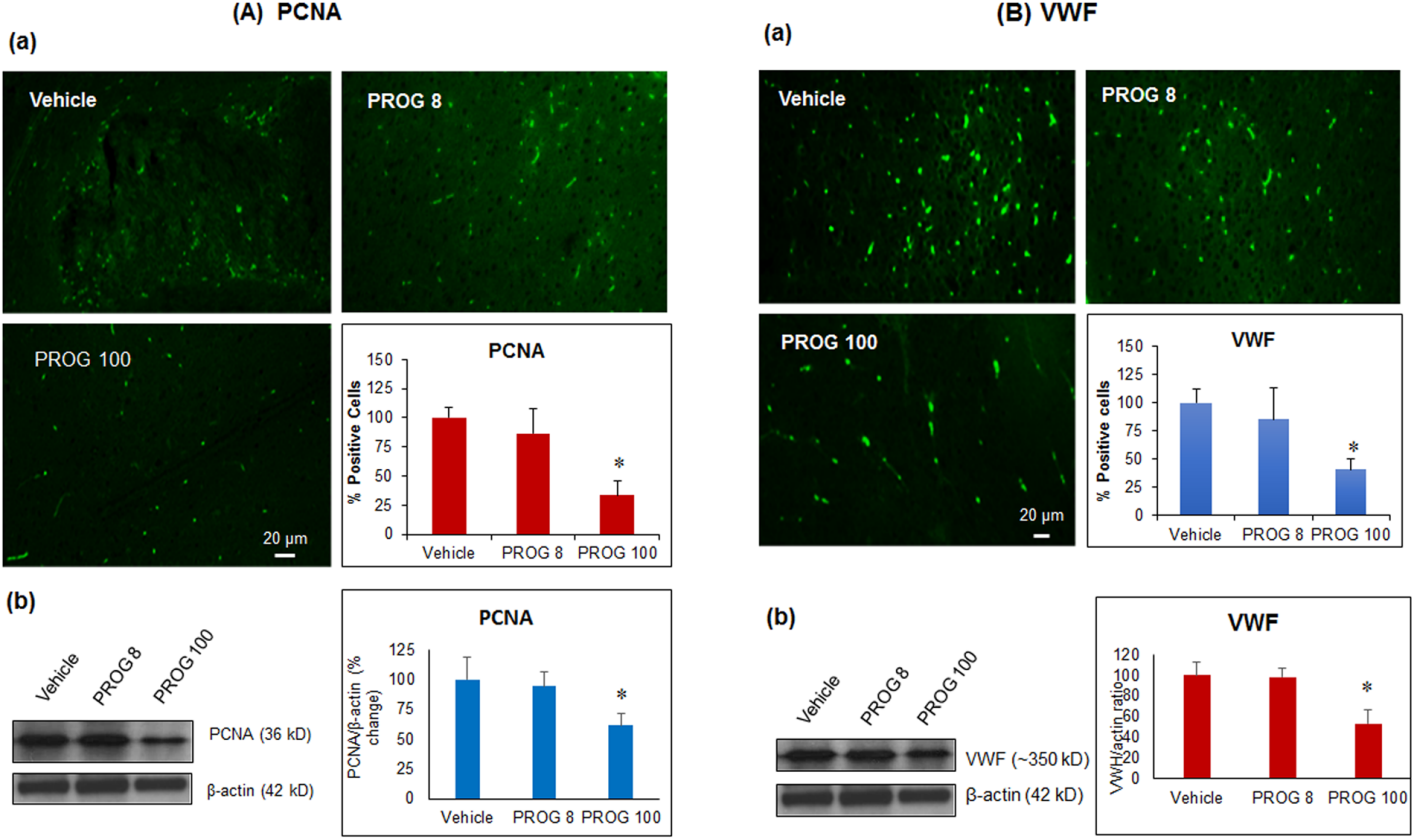
*In vivo* effect of progesterone on markers of (**A**) proliferation and (**B**) angiogenesis in tumor tissue. Representative photomicrographs of IHC and cell counting (a) and representative western blot bands with densitometric analysis (b) from different groups. Values are expressed as mean ± SD in different groups (n = 8 each). Significant difference: *P < 0.01 compared to control. For both PCNA and VWF proteins, bands were cropped from different parts of the same gel (Supplementary Figure S1).

**Figure 3:**
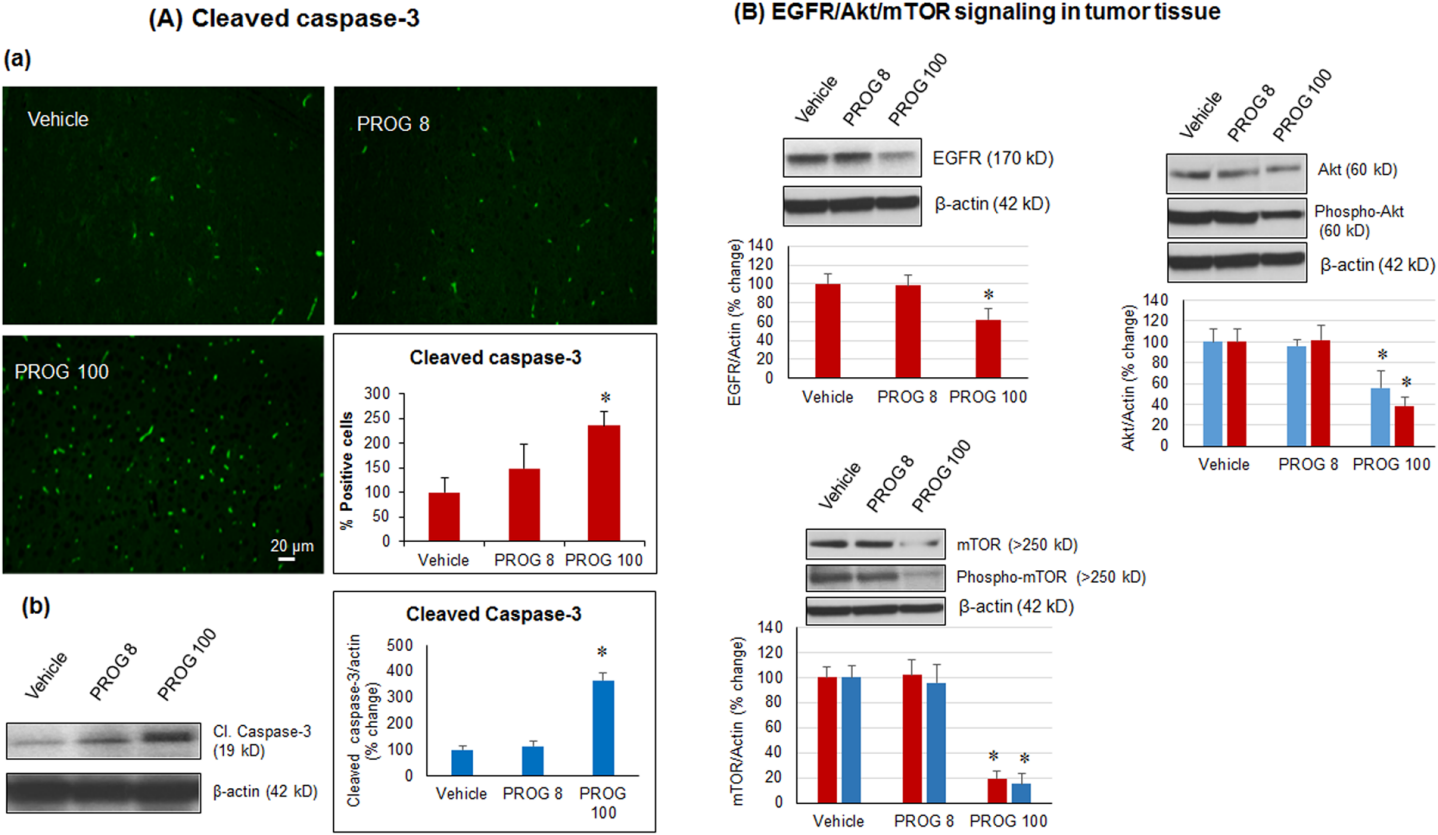
*In vivo* effect of progesterone on markers of (**A**) apoptosis and (**B**) PI3K/Akt/mTOR signaling in tumor tissue. Representative photomicrographs of IHC and cell counting (a) and representative western blot bands with densitometric analysis (b) from different groups. Values are expressed as mean ± SD in different groups (n = 8 each). Significant difference: *P < 0.01 compared to control. Bands were cropped from different parts of the same gel, or from different gels (Supplementary Figure S2).

In tumor tissue, we performed immunohistochemistry and confirmed the findings by western blot, to determine the effects of progesterone on markers of tumor proliferation (PCNA), angiogenesis (VWF), and apoptosis (cleaved caspase-3). On day 28 post-inoculation, both methods showed a significant decrease (*p* < 0.05) in PCNA (Fig. 2A) and VWF (Fig. 2B) and a significant increase (*p* < 0.05) in cleaved caspase-3 expression in the PROG100 group compared to the vehicle-treated animals (Fig. 3A). We noticed a similar but non-significant response in the PROG8 group when assessed by IHC, whereas western blot data showed no significant difference in the expression of these markers between the vehicle and PROG8 groups at day 28 post-inoculation. No significant differences were observed in the expression levels of EGFR, Akt, phospho-Akt, mTOR or phospho-mTOR between the PROG8 and the vehicle groups at day 28 post-inoculation. However, western blot revealed a significant decrease (*p* < 0.05) in the level of EGFR, Akt, phospho-Akt, mTOR and phospho-mTOR expression in the PROG100 group compared to vehicle (Fig. 3B).

### Behavioral outcomes: Progesterone improves spontaneous locomotor activity of tumor-bearing mice

**Figure 4:**
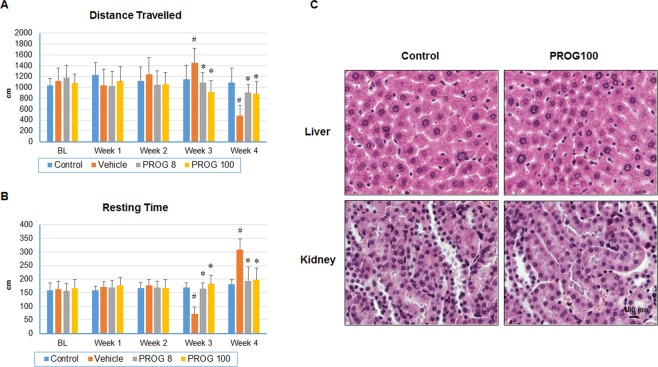
Progesterone improves spontaneous locomotor activity deficits without any organ toxicity in tumor-bearing mice. (**A**) Distance travelled; (**B**) resting time in different groups (n = 16); (**C**) high-dose progesterone toxicity in liver and kidney. Values are expressed as means ± SD. *P < 0.01: Significant difference compared to control group.

Quality of life is a major concern for cancer patients and their caregivers, especially after receiving chemo and radio therapy. We compared the spontaneous locomotor activity of mice before and after tumor establishment as a measure of the animals’ general well-being with or without progesterone treatment. Overall, PROG-treated animals showed less severity of “sickness” behaviors than vehicle-treated animals. A significant group effect was observed in total distance (F_(3,52)_ = 12.29; *p* < 0.05) and resting time (F_(3,52)_ = 16.48; *p* < 0.05) (Fig. 4).

#### Total distance

No significant difference was observed in total distance travelled among any groups at either week 1 or week 2 after tumor cell inoculation (Fig. 4A). A significant (*p* < 0.05) increase in total distance moved was observed in the vehicle group at week 3 compared to control and progesterone-treated groups. There was no significant difference between the healthy controls and either of the progesterone-treated groups at week 3. The vehicle group showed a significant decrease (*p* < 0.05) in total distance travelled at week 4 compared to control or progesterone-treated groups. There was no significant difference in deficit severity between progesterone-treated and control groups at week 4, suggesting a beneficial effect of progesterone on functional deficits.

#### Resting time

We observed no significant difference in resting time between any groups at either week 1 or week 2 after tumor cell inoculation (Fig. 4B). At week 3, the vehicle-only group showed a significant (*p* < 0.05) decrease in resting time compared to control or progesterone groups, whereas progesterone-treated groups were not significantly different from the control group. At week 4, in the vehicle group there was a significant increase in resting time compared to control or progesterone-treated groups. No significant difference in resting time was observed between progesterone-treated and control groups at week 4, suggesting a beneficial effect of progesterone on functional deficits.

### High-dose progesterone exerts no toxicity in the liver and kidney

Organs of intracranial U87MG-luc tumor-bearing mice receiving high-dose progesterone (100 mg/kg for 40 days) were collected and toxicity was assessed with H&E staining. Tissue sections from the liver and kidneys were examined for signs of acute or chronic toxicity. In the kidney, there was no acute tubular necrosis and the liver showed no evidence of centrilobular necrosis (Fig. 4C) after high-dose progesterone treatment compared to the control group (untreated, non-tumor-bearing). The two organs also showed no signs of enlargement or atrophy. Taken together, these results indicate that there was no toxicity associated with repeated, subcutaneously administered, high-dose progesterone.

### Effect of progesterone on metabolic regulators and ATP levels in GBM cells *in vitro*

**Figure 5:**
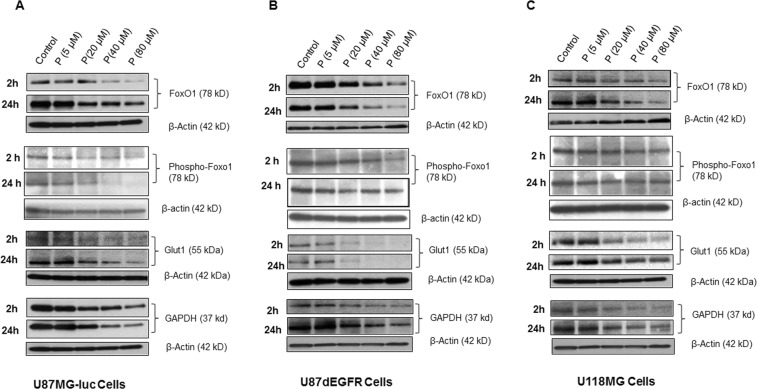
Effect of high-dose progesterone on markers of glycolytic metabolism in (**A**) U87MG-luc, (**B**) U87dEGFR, and (**C**) U118MG cells *in vitro*. Representative blots from different groups. Values are expressed as mean ± SD in different groups (n = 8 each). Significant difference: *P < 0.01 compared to control. Bands were cropped from different parts of the same gel, or from different gels (Supplementary Figure S3).

**Figure 6:**
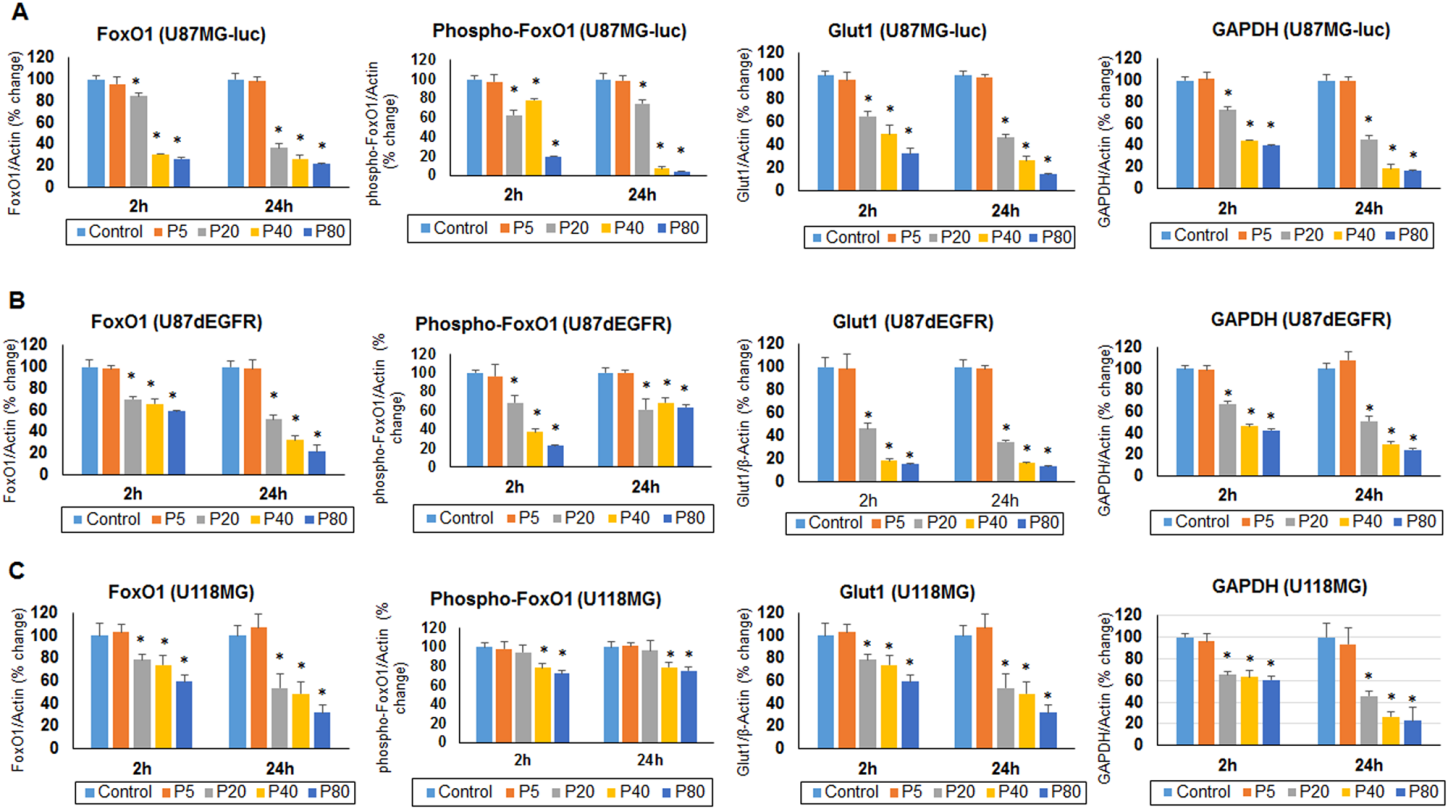
Effect of high-dose progesterone on the markers of glycolytic metabolism in (**A**) U87MG-luc, (**B**) U87dEGFR, and (**C**) U118MG cells *in vitro*. Densitometric analysis of blots (from Fig. 5) from different groups. Values are expressed as mean ± SD in different groups (n = 8 each). Significant difference: *P < 0.01 compared to control.

Preference for glycolysis over mitochondrial oxidation supports high anabolic activity of cancer cells, which results in uncontrolled proliferation, migration, invasion and metastasis. We tested the hypothesis that progesterone inhibits GBM growth by modulating the metabolic activity of GBM cells. Using U87MG-luc, U87dEGFR and U118MG (grade IV GBM) human cell lines, we examined the effect of progesterone treatments on the expression of Glut1, GAPDH, cytosolic FoxO1 and Phospho-FoxO1 levels, the key players in tumor cell glycolytic metabolism, at 2 and 24 h. We observed a significant decrease (*p* < 0.05) in the expression of all these proteins starting at 2 h but they remained elevated until 24 h following exposure to progesterone at 20, 40 and 80 µM concentrations. This modulatory effect was seen in all three cell lines compared to their respective control groups (Figs. 5 and 6). We found no difference in the expression of Glut1, GAPDH or cytosolic FoxO1 between the control group or low-dose (5 µM) progesterone groups at any time point. Our findings can be taken to suggest that high-dose progesterone exerts an inhibitory effect on the regulators of glycolytic metabolism in GBM cells.

**Figure 7:**
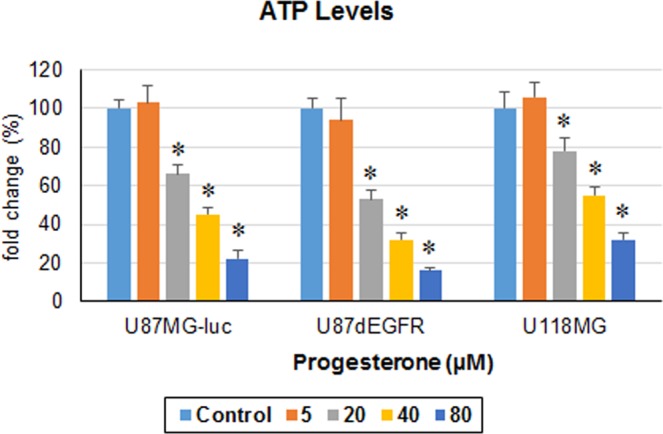
Progesterone inhibits ATP levels in GBM cells *in vitro*. ATP levels in different GBM cell lines after 24 h exposure to progesterone. Values are expressed as mean ± SD in different groups (n = 8 each). Significant difference: *P < 0.01 compared to control.

As a direct measure of progesterone’s effect on GBM cell metabolism, we measured ATP levels in U87MG-luc, U87dEGFR and U118MG cell lines 24 h after a single exposure to different concentrations of progesterone. We observed a significant (*p* < 0.05) dose-dependent inhibitory effect of progesterone on ATP levels in all three cell lines compared to their respective control groups (Fig. 7). We found no difference in ATP levels between the control group and low-dose (5 µM) progesterone groups in any cell line.

### Progesterone induces premature senescence in GBM cells *in vitro*

**Figure 8:**
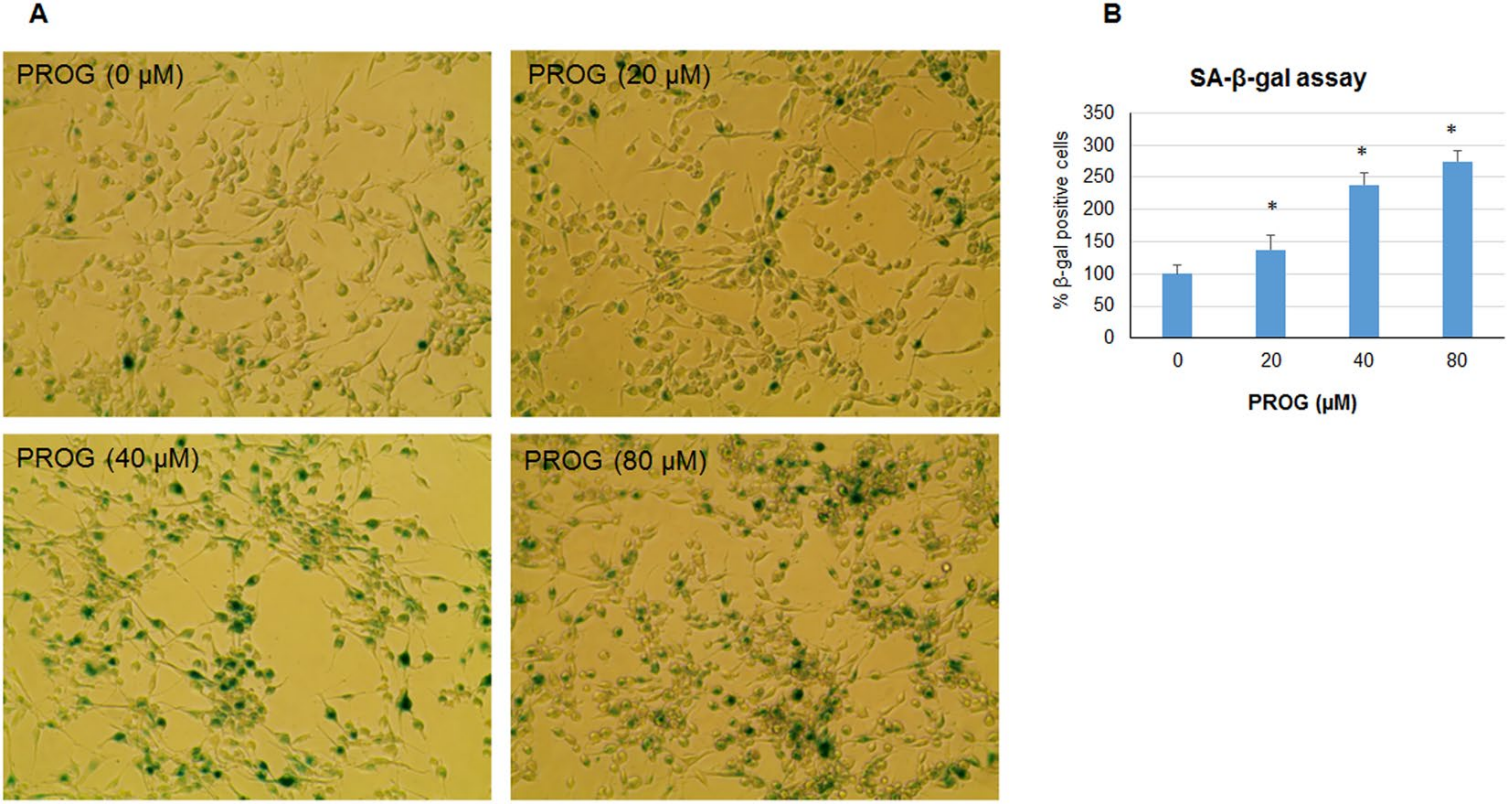
Progesterone induces premature senescence in GBM cells *in vitro*. Representative photomicrographs (**A**) and cell counting of SA-β-gal positive cells (**B**) in different groups. Values are expressed as mean ± SD in different groups (n = 8 each). Significant difference: *P < 0.01 compared to control.

Accumulation of endogenous SA-β-gal is the most common marker of cellular senescence. We measured SA-β-gal accumulation in U87MG-luc cells after 3 days of repeated exposure to progesterone (Fig. 8). We found a significant (*p* < 0.05) increase in β-gal-positive cells in progesterone-treated groups compared to the vehicle group. This senescence-inducing effect was found to be dose-dependent. The highest accumulation of β-gal was observed in the mice treated with 80 µm of progesterone compared to other doses and the vehicle group. We interpret these findings to suggest that high-dose progesterone can induce premature senescence in GBM cells.

## Discussion

### Progesterone inhibits GBM growth and prolongs survival rate in an orthotopic mouse model without any toxic side effects

**Figure 9:**
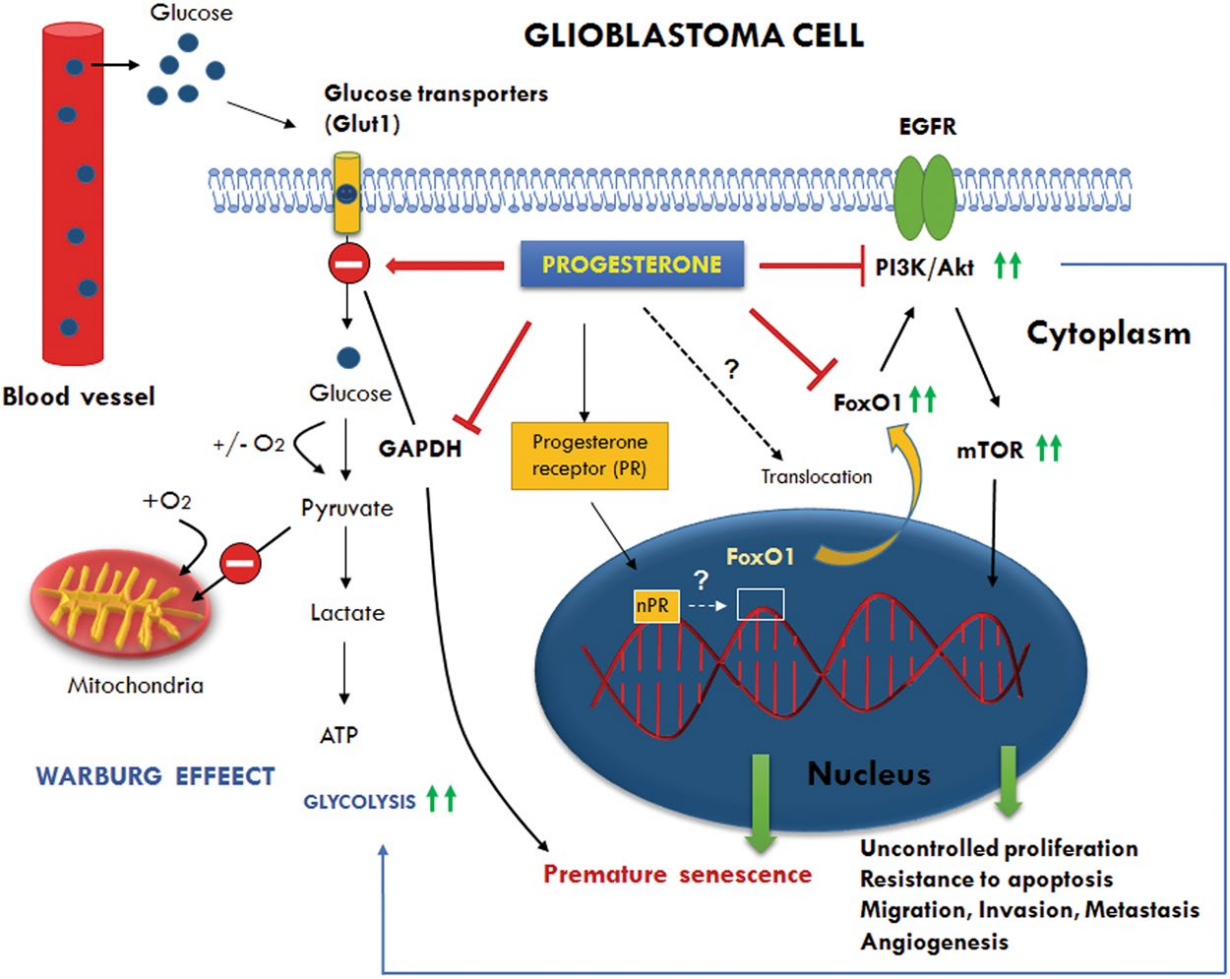
Schematic representation of the modulatory effects of progesterone on glycolytic metabolism, PI3K/Akt/mTOR signaling, and FoxO1 transcription factor in GBM. The figure shows the preference for glycolysis over mitochondrial oxidation despite sufficient levels of oxygen (the Warburg effect) which supports high anabolic activity of cancer cells for uncontrolled proliferation, migration, invasion and metastasis. Glut1 (Glucose transporter isoform 1) facilitates glucose transport into the cytoplasm and its upregulation is observed in different cancers including GBM. GAPDH (Glyceraldehyde 3-phosphate dehydrogenase) is a glycolysis enzyme whose overexpression is positively correlated with tumor progression in many cancers. Depletion of GAPDH is reported to induce senescence in tumor cells. FoxO1 (Forkhead box family O1) is a transcription factor which is critical for the regulation of cell cycle exit and arrest at G1, and induction of apoptosis. FoxO1 deregulation/inactivation leads to uncontrolled proliferation, and resistance to apoptosis. FoxO1 controls glycolytic metabolism through upstream PI3K/Akt/mTOR and downstream c-Myc activation. Our data showed that progesterone modulates glycolytic metabolism and induces premature senescence in GBM cells by inhibiting Glut1, GAPDH and cytoplasmic FoxO1 activity. Dotted arrows and question mark represent the modulatory effect of progesterone by an as yet unknown mechanism. How progesterone modulates nuclear FoxO1 activity or its translocation to the cytoplasm is still not known and needs to be defined.

In this study, we tested the effects of both low- and high-dose progesterone on the growth of GBM in an orthotopic mouse model. We also examined the effect of progesterone on the regulators of tumor glycolytic metabolism to suggest a possible mechanism of progesterone’s action in GBM. We found that high-dose progesterone inhibits GBM growth and glycolytic metabolism, induces premature senescence in GBM cells (Fig. 9), and prolongs the survival rate of tumor-bearing male mice.

#### High-dose progesterone is anti-tumorogenic

Why do we think progesterone could be a potential agent for GBM treatment? As a gestational hormone, progesterone plays a critical role in the regulation of fetal growth, which must be well controlled for healthy development and regulated growth and differentiation. It is a well-known fact that progesterone plays a regulatory role in meiosis and mitosis by controlling the activity of cell cycle regulators (cyclins, CDKs, p21^Cip1^ and p27^Kip1^)^20^. Interestingly, a lower incidence of maternal breast cancer, and long-term protection against breast cancer have been associated with high levels of progesterone during pregnancy^7^. A substantial literature supports the anti-proliferative and apoptotic effects of progesterone on breast, endometrial, ovarian, colon and salivary gland tumor *in vitro* and *in vivo*^21–25^. A recent study also suggests that high-dose progesterone prevents high-grade serous ovarian cancer *in vitro* (100 µM) and *in vivo* (5 mg/kg)^26^. We also earlier reported an antitumor effect of high-dose progesterone in human GBM cells^8,9^. We and others have shown that progesterone has anti-proliferative and apoptosis-inducing effects in other types of tumors in addition to GBM *in vitro* and *in vivo*^8,9,25,27^.

#### Progesterone inhibits proliferation and angiogenesis and induces apoptosis *in vivo*

Our IHC and protein expression data from our tumor tissue suggest an inhibitory effect of high-dose progesterone on GBM proliferation, angiogenesis, and induction of apoptosis at day 28 post-inoculation. It is also worth noting that low-dose progesterone showed some reduction in tumor volume at day 28, but it was not statistically significant. Our results suggest that it would be most prudent to use high-dose progesterone administration rather than a lower-dose treatment regimen.

The PI3K/Akt/mTOR signaling pathway is known to be highly active in GBM. It plays a critical role in drug resistance by facilitating tumor proliferation and angiogenesis, and inhibiting apoptosis even after chemo or radiotherapy^28–31^. We found high expression levels of phospho-Akt and mTOR in the vehicle group, which supports our observations of increased proliferation and angiogenesis in that group. The progesterone-treated group showed significantly lower expression of Akt, phospho-Akt, mTOR and phospho-mTOR in tumor tissue compared to vehicle controls. This inhibitory effect of progesterone on PI3K/Akt/mTOR signaling correlates with the observed decreased levels in markers of proliferation and angiogenesis.

#### Progesterone improves the quality of life of tumor-bearing mice

We recorded spontaneous locomotor activity of tumor-bearing mice at different time points to evaluate the effect of the growing intracranial GBM tumor and the effects of progesterone treatment on behavioral outcomes associated with sickness behaviors. We observed that with increasing intracranial GBM growth, mice in the vehicle group started to show functional decline compared to their non-tumor-bearing counterparts. At week 3, the vehicle-treated animals were found to be more restless or hyperactive, as evidenced by distance travelled and resting time tests. In contrast, progesterone-treated mice showed activity comparable to that of the non-tumor-bearing mice. At week 4, when the tumor size was larger, the vehicle group mice became very weak, lost body weight (data not shown), and were less mobile. Interestingly, progesterone-treated mice fared much better and their activity level was similar to that of the non-tumor-bearing mice. We think that the growing tumor led to the behavioral sickness behaviors, probably because of increasing intracranial pressure, cerebral edema and chronic inflammation. It is worth noting here that progesterone has been shown to improve behavioral deficits in a number of brain injury models including stroke and traumatic brain injuries^32,33^.

### Progesterone inhibits the modulators of glycolytic metabolism and ATP production and induces premature senescence in GBM cells

As one of the possible mechanisms of action in helping to control GBM, we tested the effect of progesterone treatment on the expression of the key regulators of glycolytic metabolism Glut1, GAPDH, FoxO1 and phospho-FoxO1, in U87MG-luc, U87dEGFR and U118MG cell lines. Also, as a direct measure of progesterone’s effect on GBM metabolism, we measured ATP levels in these cell lines. Glut1 facilitates the transport of glucose into the cytoplasm and is highly upregulated in several cancers including GBM^16^. Overexpression of GAPDH is positively correlated with tumor progression in many cancers and its depletion is reported to induce premature senescence in tumor cells^34,35^. Our data can now be interpreted to suggest an inhibitory effect of high-dose progesterone on Glut1, GAPDH expression and ATP levels in different GBM cells.

FoxO1 plays a role in the regulation of cell cycle exit and arrest at G1, and in the induction of apoptosis and antagonism of oxidative and cellular stress for normal cell growth^36^. High cytoplasmic FoxO1 and phosphorylated FoxO1 expression are associated with worse surgical outcome in patients with astrocytomas^37^. FoxO1 phosphorylation/inactivation has been associated with disease progression in several cancers^38–42^, but its clinical and pathological significance in GBM has not been fully investigated. Our data show an inhibitory effect of high-dose progesterone on cytoplasmic FoxO1 protein expression and its phosphorylation in different GBM cell lines. However, detailed mechanistic rescue experiments for FoxO1 expression in relation to progesterone treatment are needed to define the role of FoxO1 in progesterone-mediated premature senescence in GBM cells.

PI3K/Akt/mTOR signaling is known to be highly active in GBM cells, supporting proliferation, invasiveness and resistance to cell death. mTOR is also reported to control glycolytic metabolism in GBM through FoxO1 acetylation and upregulation of c-Myc in an Akt-independent manner^17^. We have previously reported that high-dose progesterone inhibits PI3K/Akt/mTOR signaling in GBM *in vivo*^8^, and our current data also show an inhibitory effect of progesterone on FoxO1, phospho-FoxO1, Glut1, GAPDH and PI3K/Akt/mTOR expression in GBM cells.

### Low-dose progesterone

In the present study we also tested the effect of a low dose (8 mg/kg) of progesterone on tumor growth *in vivo* in addition to a high dose (100 mg/kg), thinking that it could be proliferative. Interestingly, the 8 mg/kg dose also showed an anti-tumor effect during the first two weeks after tumor establishment when the tumor was smaller in size in both vehicle and progesterone treatment groups. However, by the third week, the low dose lost effectiveness in suppressing tumor growth, and tumor volume was not statistically different from that seen in the vehicle group. However, no acceleration in tumor growth was observed after low-dose progesterone treatment throughout the study. We take these findings to suggest that progesterone may not necessarily accelerate GBM growth *in vivo* even with a dose as low as 8 mg/kg; however, it is possible that even lower doses could be proliferative.

We previously reported a biphasic response of progesterone in which a low dose (nM range: 0.1–5 nM) may be proliferative but a high dose (µM range: 20–80 µM) induces cell death in different GBM cells *in vitro*^8,9^. Similar to our findings, a recent report on berberine showed cell proliferation at low doses (1.2–5 μM) and inhibited cell proliferation at high doses (10~80 μM) in several cancer cell lines^43^. Additionally, several molecules used in anticancer therapy elicit a bell-shaped dose-response, for example, bortezomib^44^, HMG-CoA reductase inhibitors^45,46^, plasminogen activator-1 (PAI-1)^47^, RGD-mimetic integrin inhibitors^48,49^, TGF-β1^50^, and TGF-β3^51^. These molecules are reported to stimulate tumor angiogenesis at low concentrations, while inhibiting angiogenesis at higher doses. It is essential to emphasize that the reported proliferative doses are far lower than those used in our studies. Taken together, the findings underline the critical importance of dose selection of progesterone or any other chemotherapeutic agent and suggest that only a confirmed high dose of progesterone should be used in any chemotherapeutic study. Therefore, going forward in seeking safe and effective treatments for GBM, it will be of critical importance to select the right dose of any potential therapeutic agent.

### Conclusions, potential limitations and future directions

Our findings suggest that high-dose progesterone treatment inhibits GBM growth and prolongs survival rate in mice without exerting any organ toxicity. Our findings also indicate that high-dose administration of progesterone could have a modulatory role in metabolic reprogramming in GBM cells (Fig. 9). However, we recognize the need for doing direct metabolic assays like metabolic flux or mitochondrial respiration assays by Seahorse XF96 analyser to obtain a more direct evidence of progesterone’s effect on GBM cell metabolism. Now, with data showing progesterone inhibition of tumor growth, it makes it reasonable to perform more detailed mechanistic studies to help define the interaction of nuclear progesterone receptor with nuclear FoxO1 activity in GBM. This can be done by targeting nuclear FoxO1 activity either pharmacologically or by using a knockout, immunosuppressed mouse model when it becomes available. Further studies should also be done to determine whether the effects we have observed here will also be seen in females. Taken all together, we think that high-dose progesterone treatment might be a useful adjunct to current standard of care therapies used to control the tumor growth.

## Materials and Methods

For the *in vivo* study, we calculated the starting sample sizes and power needed to reject the null hypothesis (H_0_) of no effects on tumor growth with a *p*-value of 0.05 at a power of 0.8, and determined that we needed 7 mice per group. We conducted two independent experiments with n = 8/group/experiment and pooled the data (n = 16) for the final analysis. All tumor volume measurement, drug treatment, and western blot and immunohistochemical assays were performed independently by a researcher blinded to the experimental conditions (i.e., treatment versus vehicle alone). None of the animals that survived tumor inoculation and drug treatments were excluded from the experiments. The experiments were performed in accordance with the ARRIVE guidelines.

### Cell culture

A human GBM cell line, U118MG, was purchased from ATCC (Manasses, VA). The genetically modified human GBM cell line U87dEGFR, which stably expresses the EGFRvIII mutant form of EGFR, was received as a generous gift from Prof. Erwin Van Meir (Department of Neurosurgery, Emory University). A firefly luciferase stable U87MG cell line (U87MG-luc) was received as a generous gift from Dr. Theodore Nicolaides (Department of Pediatrics and Neurosurgery, University of San Francisco, CA). Cells were grown in DMEM culture medium (4.5 gm glucose) supplemented with 10% fetal bovine serum and 1% antibiotic and antimycotic solution at 37 °C in a 5% CO_2_ environment.

### Luciferase assay in U87MG-luc cells

U87MG-luc cells were cultured in flasks until they were sub-confluent. For the luciferase assay, 0, 50,000 and 100,000 cells were seeded in a 96-well plate. The substrate D-luciferin was added to the cells and the bioluminescence was recorded using the IVIS imaging system spectrum at an Emory core facility.

### Cell viability assay in U87MG-luc cells

To test the sensitivity of U87MG-luc cells to progesterone, these cells were seeded (0.5 × 10^5^/well) in a 24-well plate and kept under starvation overnight prior to drug exposure. Progesterone (P3972, Sigma Chemicals, St. Louis, MO) stock was prepared in absolute dimethyl sulfoxide (DMSO) and further diluted in culture medium. The final concentration of DMSO was kept at <5 µl/ml. Cells were exposed to different concentrations of progesterone (0.1, 1, 5, 10, 20, 40, 80 µM) as repeated exposure for 3 and 6 days. For repeated exposure, culture medium was replaced daily and each of the progesterone doses was added to the cells every day. Cell viability was assessed by 3-(4,5-dimethylthiazol-2-yl)-2,5-diphenyltetrazolium bromide (MTT) reduction assay. The reaction is based on the cleavage of the tetrazolium ring of the pale yellow MTT into dark blue formazan crystals by mitochondrial dehydrogenase enzyme in viable cells. Formazan crystals accumulate within the cells due to their impermeability to the cell membrane and are then solubilized by adding DMSO (50 ml). The intensity of blue-colored formazan solution is directly proportional to the number of surviving cells. Concentrations were determined by photometric analysis. Briefly, 20 μl of MTT solution (5 mg/ml phosphate buffered saline (PBS)) was added per well and incubated at 37 °C for 4 h until a purple precipitate was visible. DMSO (500 μl) was added to solubilize the crystals and the absorbance was read at 570 nm.

### Protein expression studies by western blot

For *in vivo* studies, protein was extracted from tumor tissue using T-per extraction buffer (Pierce, Rockford, IL) with protease inhibitors, and assayed for protein concentration by BCA (bicinchoninic acid assay) microplate protein assay (Pierce, 23225). For *in vitro* studies, tumor cells (U87MG-luc, U87dEGFR and U118MG) were seeded (0.5 × 10^6^) in petri dishes and kept under starvation overnight prior to progesterone or vehicle (DMSO) exposure. Cells were exposed to different concentrations of progesterone (0, 1, 5, 40 and 80 µM) for 24 h. Protein was isolated using the RIPA lysis buffer system (sc-24948A, Santa Cruz Biotechnology, Santa Cruz, CA) and assayed for protein concentration by BCA microplate protein assay (23225, Pierce, Waltham, MA). Western blot assay was used to examine the effect of progesterone on protein expression in different GBM cell lines and tumor tissue. Protein samples (50 µg) were separated under reducing and denaturing conditions by 4–20% acrylamide Criterion gel (BioRad, Hercules, CA) at 200V for 1 h and transferred to a polyvinylidene difluoride (PVDF) membrane at 100V for 30 min. The non-specific binding sites of the membrane were blocked with 5% non-fat dry milk in PBS-T (PBS containing 0.05% Tween-20). The membranes were probed with the following primary antibodies overnight at 4 °C: FoxO1 (#2880 S; Cell Signaling Technology, Danvers, MA), Phospho-FoxO1 (Ser256; #9461; Cell Signaling Technology, Danvers, MA), Glut1 (sc377228; Santa Cruz Biotechnology), GAPDH (sc365062; Santa Cruz Biotechnology), cleaved caspase-3 (#9664 P; Cell Signaling Technology, MA), β-Actin (AC74; Sigma Chemicals, St. Louis, MO), proliferating cell nuclear antigen (PCNA; sc9857; Santa Cruz Biotechnology), Von Willebrand factor (VWF; sc365712; Santa Cruz Biotechnology), epidermal growth factor receptor (EGFR; sc03; Santa Cruz Biotechnology), Phospho-Akt (Ser473; Cell Signaling Technology), mTOR (#32028; Abcam Inc. Cambridge, MA), Phospho-mTOR (59.Ser 2448; sc-293133; Santa Cruz Biotechnology). Membranes were then incubated in their respective horseradish peroxidase (HRP) -conjugated secondary antibodies. Blots were developed using a chemiluminescent substrate (Pierce) for 5 min. Chemiluminescent bands were detected on a Kodak autoradiography film in a darkroom and their densities were measured using NIH ImageJ software. In each figure, representative blot images were selected from the same gel.

### ATP assay

U87MG-luc, U87dEGFR and U118MG cells were cultured and treated with different concentrations of progesterone for 24 h. ATP assay was performed using an ATP assay Kit (MAK190; Sigma Chemicals) as per the manufacturer’s instructions.

### Senescence-associated β-galactocidase (SA-β-gal) activity assay

U87MG-luc cells were cultured and repeatedly treated with the different concentrations of progesterone or vehicle (DMSO) daily for 3 days. On day 4, cells were washed, fixed and stained for SA-β-gal activity using the Senesence β-Galactosidase Staining Kit (Cell Signaling Technology, #9860) according to the manufacturer’s instructions.

### Intracranial inoculation of U87MG-luc cells in nude mice

The *in vivo* chemotherapeutic effect of progesterone on luciferase-stable U87MG-luc xenografts was studied in adult male athymic nude mice (Hsd: Athymic Nude-*Foxn1nu*; Harlan, Indianapolis, IN). Protocols (DAR-2002754-ENTRPR-N) were approved by the Institutional Animal Care and Use Committee (IACUC) of Emory University. Briefly, U87MG-luc cells (3 × 10^6^/5 µl culture medium) were stereotactically injected into the brains of the mice. Animals were anesthetized by i.p. injection of ketamine (80 mg/kg)/xylazine (10 mg/kg) mixture and then secured to a stereotactic frame. Body temperature (~37 °C) was maintained by a heating pad. A paramedian incision was made, and a 1.5-mm burr hole was drilled 1 mm anterior to the coronal suture on the right hemisphere and 2 mm lateral from the midline. A Hamilton syringe was used to inject U87MG-luc cells into the posterior right frontal lobe of the brain over a period of 2 min. The burr hole was filled with ostene (a bone hemostasis agent), the skull was washed with sterile water to destroy any cells leaked into the subgaleal space, and the scalp was closed with 3–0 running vicryl stitches. The tumor cells were allowed to grow for 6 days after inoculation and before any progesterone treatments were applied.

### Bioluminescent Imaging (BLI) for monitoring tumor growth

Mice were anesthetized using a ketamine/xylazine mixture (100 mg/ml; 2:1 ratio) and injected with the luciferin substrate. Tumor establishment was confirmed by BLI on day 7 using an IVIS imaging system at the Emory University imaging core facility. Mice were imaged once every week after the tumors were established and the treatment was started.

### Progesterone treatment

Progesterone (P3972, Sigma) was prepared in sterile 30% 2-hydroxypropyl-beta-cyclodextrin (HBC; H107, Sigma) solution for *in vivo* experiments. After confirming tumor establishment, mice were randomly divided into three groups (n = 16 each): Vehicle (HBC); progesterone (8 mg/kg, PROG 8); and progesterone (100 mg/kg, PROG 100). An additional group (n = 8) of untreated, non-tumor-bearing nude mice served as controls to assess drug toxicity based on spontaneous locomotor activity, survival, and body weight. A s.c. injection of either progesterone or vehicle alone was given daily at the same approximate time for 28 days. On day 29, all vehicle-treated mice were euthanized because they showed the maximum permitted tumor burden according to Emory IACUC guidelines. Eight mice from each progesterone-treated group were euthanized along with the vehicle group. The remaining 8 mice/group were kept alive for survival studies. The endpoint criterion for the survival study was the maximum tumor burden allowed by the IACUC of Emory University. These animals continued to receive progesterone injections until they reached the tumor burden limit. They were then imaged for tumor volume using BLI.

### Spontaneous locomotor activity

Mice were tested for open field activity under red light in a quiet environment. For each trial, two animals were tested simultaneously in individual boxes using the Opto-Varimex© activity monitoring system (Columbus Instruments, Columbus, OH). For baseline activity, the mice were tested 1 day before cell inoculation followed by weekly testing for 4 weeks. We measured total distance traveled and resting time as described previously^52^.

### Histology

Mice were fatally anesthetized with isoflurane and brain, liver, and kidney were extracted and fixed in 10% buffered formalin. Fixed tissues were embedded in paraffin and sectioned at 10-µm thickness. All tissue sections were stained in Hematoxylin and Eosin (H&E) solution and coded to obscure group identity. The brains were used for measurement of tumor size. Liver and kidney were used to evaluate drug toxicity. The sections were examined with an Olympus microscope and pictures were taken using Image-Pro software^©^.

### Statistical analysis

All data were expressed as mean ± standard deviation of the mean (SD). Statistical significance was set at *p* < 0.05. All *in vitro* data were analyzed using one-way ANOVA followed by Dunnett’s test. After a power analysis to determine sample size needed to observe a significant difference in tumor growth, the *in vivo* tumor data were analyzed using repeated measures ANOVA followed by an LSD *post-hoc* test. A two-tailed unpaired *t-*test was used to analyze western blot densitometry and activity data. Analyses were calculated using SPSS™ 24.0 statistical analysis software (IBM, Armonk, NY).

## Supplementary information

Supplementary Information
