# Supplementary material for: Progesterone Treatment Attenuates Glycolytic Metabolism and Induces Senescence in Glioblastoma
Source: Sci Rep. 2019 Jan 30;9:988. doi: 10.1038/s41598-018-37399-5 (PMC6353890; doi:10.1038/s41598-018-37399-5)
Supplement: Supplementary file 1 — Supplementary Information [file 41598_2018_37399_MOESM1_ESM.docx]

**Progesterone Treatment Attenuates Glycolytic Metabolism and Induces Senescence in Glioblastoma**

**Fahim Atif*, Seema Yousuf, Claudia Espinosa-Garcia, Elena G. Sergeeva, Donald G. Stein**

Brain Research Laboratory, Department of Emergency Medicine, School of Medicine, Emory University, Atlanta, GA 30322 USA

*****Correspondence to:** Fahim Atif*,* Department of Emergency Medicine, Brain Research Laboratory, 1365B Clifton Road NE, Suite 5100, Emory University, Atlanta, GA 30322, USA. Phone: (404) 727-7614; Fax: (404) 778-2630; E-mail: [fatif@emory.edu](mailto:fatif@emory.edu)

**Supplementary Figure S1: *In vivo* effect of progesterone (PROG)** **on markers of (A) proliferation and (B) angiogenesis in tumor tissue.** Full-length gels are shown for Figure 2 (PCNA and VWF). For each protein, bands were cropped from the same respective part (indicated by the red arrow) of the same gel.

**Supplementary Figure S2: *In vivo* effect of progesterone (P)** **on markers of (A) apoptosis and (B) PI3K/Akt/mTOR signaling in tumor tissue.** Full-length gels are shown for Figure 3. Bands were cropped from different parts of the same gel, or from different gels.

**Supplementary Figure S3: Effect of high-dose progesterone (P) on the markers of glycolytic metabolism in (A) U87MG-luc (B) U87dEGFR and (C) U118MG cells *in vitro*.** Full-length gels are shown for the main Figure 5 (Western blot) and Figure 6 (densitometry). Bands were cropped from different parts of the same gel, or from different gels.
